# The Association Between Possible Stressors and Mood Outcomes in Older Residents of Long-Term Care Facilities

**DOI:** 10.3389/fpsyt.2022.811252

**Published:** 2022-04-04

**Authors:** Milou J. Angevaare, Hein P. J. van Hout, Martin Smalbrugge, Annette H. Blankenstein, Cees M. P. M. Hertogh, Jos W. R. Twisk, Karlijn J. Joling

**Affiliations:** ^1^Amsterdam UMC, Vrije Universiteit Amsterdam, Department of Medicine for Older People, Amsterdam Public Health Research Institute, Amsterdam, Netherlands; ^2^Amsterdam UMC, Vrije Universiteit Amsterdam, Department of General Practice, Amsterdam Public Health Research Institute, Amsterdam, Netherlands; ^3^Department of Epidemiology and Data Science, Amsterdam Public Health Research Institute, Amsterdam UMC, Vrije Universiteit Amsterdam, Amsterdam, Netherlands

**Keywords:** resilience, mood outcome, self-report, stressor, conflict, major life stressor, LTCF, nursing home

## Abstract

**Introduction:**

Resilience incorporates the presence of a positive response to some type of stressor. To properly explore resilience, it is important to systematically identify relevant stressors. We aimed to identify (combinations of) stressors with the strongest relationship with observer-reported and self-reported mood outcomes in older residents of long-term care facilities (LTCFs) in The Netherlands.

**Materials and Methods:**

We included 4,499 older (≥60) residents of 40 LTCFs who participated in the Dutch InterRAI-LTCF cohort between 2005 and 2018. The association of possible stressors (single stressors, number of stressors, and combinations of two stressors) in this population with observer-reported (Depression Rating Scale) and self-reported mood outcomes was analyzed using multilevel tobit models and logistic regressions.

**Results:**

Major life stressor [“experiences that (threatened to) disrupt(ed) a person's daily routine and imposed some degree of readjustment”] and conflict with other care recipients and/or staff were most strongly associated with both mood outcomes. Furthermore, conflict was a particularly prevalent stressor (24%). Falls, fractures, and hospital visits were more weakly or not associated at all. Overall, the associations were similar for the mood outcomes based on observer-report and self-report, although there were some differences. Multiple stressors were more strongly associated with both mood outcomes than one stressor.

**Conclusion:**

Major life stressor and conflict emerged as important stressors for resilience research within the psychological domain in LTCF residents. Further (longitudinal) research is necessary to determine the directionality and relevance of the strong association of conflict with mood for LTCF practice.

## Introduction

In recent years, the concept of resilience has gained popularity in aging research. This concept encompasses the presence of a positive response (outcome) to some type of stressor (adversity) and the mechanism by which that positive response is achieved (1). However, there is some scientific discussion on what a stressor should entail (2, 3).

One perspective is that a stressor should ordinarily “pressure adaptation and lead to negative outcomes in a majority of people” (4, 5). An earlier study operationalized this perspective in community-dwelling older adults by requiring a significant negative relationship with quality of life for each stressor included in their resilience analyses (6). This is a uniquely thorough approach as the choice of stressor is often not substantiated in resilience research (2). Although stressors have been explored in community-dwelling older adults using both quantitative and qualitative research methods, to our knowledge, this has not been done in older adults living in long-term care facilities (LTCFs) (6, 7). To properly explore resilience in LTCFs, it is important to systematically identify stressors this population as well.

There are several aspects to take into account when studying stressors in the context of resilience. Hildon et al. showed that stressors often cluster within an individual and that a combination of stressors had a greater impact on quality of life than one stressor (2, 6). Investigating the combinations of stressors may be of extra importance in older adults as there is an abundance of possible stressors in old age, such as personal illness, illness or death of relatives, and cognitive impairment (1).

In addition, a common perspective is that resilience is not generalizable across domains (2). Therefore, when identifying relevant stressors for resilience research, the relationship with the specific outcome domain of interest should be explored. The majority of resilience research in older adults has occurred within the psychological domain. In most of these studies, the outcome has been defined as the absence of psychological distress as operationalized by the absence of depressive/mood symptoms (8). Given the relation between mood symptoms and quality of life, investigating resilience, and thus stressors, in relation to mood symptoms can play a great role in identifying factors that can reduce the burden of these stressors (9).

Furthermore, it is important to consider that the meaning of a possible stressor for the person experiencing this stressor can be different from the meaning assigned by an observer/researcher/professional to the stressor (1, 10, 11). It has therefore been proposed that an ideal resilience research design includes both objective and subjective outcomes (4).

The aim of this study is to identify (combinations of) stressors with the strongest relationship with observer-reported (objective) and self-reported (subjective) mood outcomes in older residents of LTCFs in The Netherlands.

## Materials and Methods

### Study Design

In this cross-sectional study, the association of possible stressors with observer-reported and self-reported outcomes was determined in older residents of LTCFs using the Dutch InterRAI-LTCF cohort.

### Data and Population

Analyses were conducted with assessments of residents (≥60 years) of LTCFs throughout The Netherlands using the interRAI LTCF assessment instrument. The routine care assessments consist of ±250 items across 19 domains of health and functioning and are conducted by nursing staff. All items are scored by the assessors, unless stated otherwise (i.e., self-reported). The assessments were an element of the standard care for the residents of each of the participating facilities. Data collection has been described in detail previously (12).

After de-identification, data were transferred to the interRAI-LTCF database at Amsterdam University Medical Centers–location VU. Data collection occurred in compliance with European Union (EU) legislation. Since 2014 an opt-out procedure was applied in compliance with the EU General Data Protection Regulation. Residents were informed by facility staff that their data could be used for research purposes and they had the opportunity to object. The VU ethics committee approved the use of data for research in this way.

#### Assessment Selection

The data utilized for this study were collected from 2005 to 2018 and consist of a total of 29,199 assessments involving 7171 residents. Assessments from eight facilities that participated in a temporary pilot ( ≤ 15 total assessments) were excluded. Subsequently, we selected the first assessment for each resident, which met these criteria: 1. not a discharge assessment, 2. length of stay of ≥90 days, and 3. the resident was ≥60 years old. A stay of ≥90 days was required because of our interest in stressors that occurred within the LTCF setting. Discharge assessments were excluded as these are utilized to register discharge/death and are thus incomplete.

### Measures

#### Stressors

Possible stressors within the LTCF-assessment were identified in several stages.

First, six experts individually selected possible stressors relevant to this population from the complete LTCF item list based on their expertise [e.g., (elderly care) medicine and aging research]. In a subsequent group discussion, these possible stressors were narrowed down to events, whereas symptoms such as pain and dizziness were excluded. This led to eight possible stressors as described in Table 1. An association between the variable major life stressor and mood symptoms in older adults has been established previously in community-dwelling older persons (13). Conflict with other residents and staff has been shown to be associated with sadness in a Canadian sample of LTCF residents with dementia (14). The other events are health-related (such as hospitalization, fractures, and falls). The theoretical basis of including these events is that they lead to a disruption in daily routine and often functional decline, which, in turn, can affect mood.

**Table 1:** Possible stressors.

Hospital stay in the last 90 days
Major life stressor in the last 90 days
Conflict with or repeated criticism of staff and/or other care recipient
Hip fracture in the last 30 days
Other fracture in the last 30 days
Falls in the last 90 days (1 or more)
Inpatient acute care in hospital with overnight stay in the last 90 days
Emergency room visit in the last 90 days

The stressor hospital stay was based on the interRAI variable “time since last hospital stay”. This variable was dichotomized: indicating the occurrence of any type of hospital stay (both planned and acute) in the previous 90 days.

The dichotomous (yes/no) variable “major life stressors in the last 90 days” is defined by the interRAI manual as “experiences that either disrupted or threatened to disrupt a person's daily routine and that imposed some degree of readjustment”. Several examples are provided, such as the death or severe illness of a close family member or friend (15).

The stressor conflict was based on two items, namely, “conflict with or repeated criticism of staff” and “…other care recipients”. These dichotomous items are described as the presence of “a reasonably consistent pattern of hostility or criticism directed toward one or more staff” and “other care recipients”, respectively, over the last 3 days. We created a dichotomous variable indicating conflict with staff and/or other care recipient.

The stressors hip fracture and other fracture are based on the interRAI items “hip fracture during last 30 days” and “other fracture during last 30 days”. These items were dichotomized into two variables indicating the presence of the diagnosis hip fracture/other fracture in the previous 30 days.

The interRAI item “falls” with four categories ranging from no falls in last 90 days to two or more falls in last 30 days was dichotomized into a variable indicating the occurrence of falls in the last 90 days.

For the acute hospital care stressors, the interRAI items “inpatient acute care hospital with overnight stay” and “emergency room visit (not counting overnight stay)” were used. These were recoded into two dichotomous variables indicating the occurrence of the specifically acute hospital stay and emergency room visit, respectively, in the previous 90 days.

#### Outcomes

The association of the possible stressors with two mood outcomes was explored:

1. The Depression Rating Scale (DRS) represents observer-reported mood symptoms. It is based on seven observed mood symptoms: made negative comments; persistent anger with self or others; expression of unrealistic fears; repetitive health complaints; repetitive anxious complaints/concerns (non-health–related); sad, pained, worried facial expression; and crying/tearfulness. Each of these items is scored 0 to 3 with 0 indicating that the symptom is not present and 3 indicating that the symptom is present every day for the last 3 days. These are recoded to three categories: not present, present up to 2 of the last 3 days, and present every day of last 3 days. The total score ranges from 0 to 14, with 14 indicating that all mood symptoms were present during the last 3 days (16). In a sample of 4,156 residents in seven EU countries the average weighted kappa's for test-retest and interrater reliability were 0.75 and 0.70, respectively, across all 14 interRAI mood symptoms, both observer-reported and self-reported (17). In a Korean sample of 434 residents, the kappa for interrater reliability for all (11) observer-reported mood outcomes was 0.67. The Cronbach's alpha for internal consistency of the items in the DRS in this sample was 0.82 (18).
2. Self-reported mood was measured in the LTCF assessment with three self-reported mood items: loss of interest, sadness, and anxiety. The resident is asked to report whether they have experienced these mood symptoms in the last 3 days. We created a composite score, which we call SRM, ranging from 0 to 6, in a similar fashion as the DRS. Not willing/able to respond was coded as missing. A score of 6 signifies that all three mood symptoms were present during the last 3 days. In the Korean sample, the kappa for the interrater reliability for the three self-reported symptoms was 0.72 (18).

#### Covariates

Covariates included demographics such as age, gender, and length of stay within the facility. Other covariates were indicators of health: 1. the number of a total of 15 common somatic diagnoses (neurological, cardiac/pulmonary, infections, cancer, and diabetes mellitus) and 2. the presence of a psychiatric diagnosis (anxiety, depression, and/or schizophrenia).

Several interRAI scales were used as indicators of functioning in different domains. Cognitive functioning was assessed with the Cognitive Performance Scale (CPS) including items on memory impairment and executive functioning. Scores range from 0 (intact) to 6 (very severe impairment). A score of 3 or more indicates moderate to severe impairment. The CPS has been shown to be correlated with the mini-mental state examination (MMSE) in validation studies (19, 20). Activities of daily living (ADL) functioning was represented by the ADL Hierarchy scale (ADLH), in which scores range from 0 (no impairment) to 6 (total dependence) (21). Social functioning was represented by a Revised Index of Social Engagement (RISE). Scores range from 0 to 6, with higher scores indicating greater social engagement within the facility (22).

### Analyses

Descriptive analyses revealed substantial floor effects in both the DRS and SRM (37% and 60% of the scores being 0, respectively). Therefore, the relationship between each stressor and outcome combination was explored using multilevel tobit models. Tobit models have been proven to ameliorate the problems caused by floor effects (23, 24). A latent outcome variable is estimated for those with a score of 0, representing what the outcome would have been for this individual if scores below 0 were possible. The regression line is based on the true outcome score for those with a score above 0 and the latent outcome score for those with a 0 on the true outcome score (23).

All data preparation and descriptive statistics were performed in IBM SPSS statistics version 26 (25). All analyses were performed in STATA version 14 (26).

We performed complete case analyses as there was a minimal amount of missing data for all variables, except SRM. Approximately 18% of the residents did not respond to the self-reported questions. Therefore, the models with SRM as outcome were based solely on the population that was capable/willing to respond to the questions. Using descriptive statistics (Mann–Whitney, T-, and χ^2^-tests), the differences between those with and without complete SRM were explored.

#### Single Stressor

An unadjusted and an adjusted model were generated to determine the association between each stressor and each outcome (DRS and SRM), leading to four models per stressor. The care facility was included as a second level in each model.

All covariates described above were added to the adjusted models. Gender and cognition (dichotomously, based on the cutoff for CPS of ≥3) were explored as effect modifiers by adding their interaction term with the stressors to each adjusted model. Gender differences in the experience of mood symptoms have been extensively described (27, 28). Cognitive functioning may have an effect on how stressors are experienced and, therefore, on their relationship with mood.

#### Combination of Stressors

Analogous to the single stressors analyses, four tobit models were generated in which the independent variable was the number of stressors at the time of assessment (0, 1, 2, 3, or more). Similarly, four tobit models were performed for each of the most common (at least as common as the least common single stressor) combinations of two stressors.

#### Sensitivity Analyses

To explore the robustness of the associations between stressors and outcomes, we performed unadjusted and adjusted multilevel binary logistic regressions for each of the stressor–outcome combinations described above. For these analyses, the outcomes were dichotomized. For the DRS, the standard cutoff of 3 or more, indicating clinically significant mood symptoms, was utilized for the dichotomization (16). For the SRM, a cutoff of 2 was determined on the basis of the distribution of the SRM scores relative to the DRS scores across all baseline assessments of the complete the Dutch InterRAI-LTCF cohort.

## Results

We included 4,499 residents from 40 facilities. Table 2 shows the baseline characteristics. Prevalence of the stressors ranged from 2.0% (other fracture) to 23.5% (conflict).

**Table 2:** Description of the complete study population.

**Characteristic**	***n* (Complete sample = 4,499)**	**Descriptives**
Age in years, mean (SD)	4,499	83.5 (7.74)
Women, *n* (%)	4,494	3,183 (70.7%)
LOS in days, mean (SD)	4,499	711.1 (1499.9)
Number of somatic diagnoses, mean (SD)	4,499	1.74 (1.25)
Presence of psychiatric diagnoses, *n* (%)	4,499	1,110 (24.7%)
CPS, mean (SD)	4,460	1.93 (1.70)
ADLH, mean (SD)	4,499	2.15 (1.78)
RISE, mean (SD)	4,496	3.64 (2.10)
Presence of stressor, *n* (%)		
• Conflict with staff and/or other care recipient	4,496	1,058 (23.5%)
Falls	4,499	960 (21.3%)
Major life stressor	4,496	846 (18.8%)
Hospital stay	4,499	424 (9.4%)
Inpatient acute care	4,496	292 (6.5%)
Hip fracture	4,499	155 (3.4%)
Emergency room visit	4,499	123 (2.7%)
Other fracture	4,499	91 (2.0%)
DRS, median/mean (SD)	4,497	1/2.35 (2.84)
SRM, median/mean (SD)	3,705	0/1.00 (1.56)

*CPS, Cognitive Performance Scale (range 0–6); ADLH, Activities of Daily Living Hierarchy Scale (range 0–6); RISE, Revised Index of Social Engagement (range 0–6); DRS, Depressive Rating Scale (range 0–14); SRM, Self-reported Mood scale (range 0–6)*.

Those with SRM were slightly older (83.6 vs. 82.7 years), had a shorter average length of stay (700 vs. 764 days), had less somatic diagnoses (1.7 vs. 2.0), were less cognitively impaired (CPS: 1.6 vs. 3.6), were less impaired on ADL functioning (ADLH: 1.9 vs. 3.4), had a greater level of social engagement (3.9 vs. 2.4), and had a lower score on the DRS (mean: 2.1 vs. 3.5; median score: 1 vs. 3). In addition, they were less likely to have a psychiatric diagnosis (23% vs. 31%).

### Single Stressor

Table 3 provides insight in the association between the presence of each stressor and the two outcomes. The stratified adjusted regression coefficients for those stressors that showed a statistically significant interaction with cognition or gender are provided in the Supplementary Materials (Supplementary Table E-1). All statistically significant associations in the adjusted models were positive. We will describe the results of the adjusted models, including the significant interactions with cognition and gender. Unless stated otherwise, the unadjusted results were similar.

**Table 3:** Overview of the association between single stressors and: a. observer-reported mood (DRS); b. self-reported mood (SRM) in the subpopulation with SRM complete.

**a. DRS**				
	**Unadjusted**		**Adjusted^a^**	
**Single stressor**	***n***	**Regression coefficient (95% CI)**	***n***	**Regression coefficient (95% CI)**
1. Conflict	4,496	**3.69 (3.43–3.95)**	4,454	**3.07 (2.83–3.30)**
2. Falls	4,497	**1.18 (0.88–1.48)**	4,454	**0.86 (0.59–1.12)**
3. Major life stressor	4,496	**1.58 (1.27–1.89)**	4,454	**1.46 (1.18–1.74)**
4. Hospital stay	4,497	**−0.57 (−1.01–0.13)**	4,454	−0.05 (−0.45–0.35)
5. Inpatient acute care	4,494	**−0.57 (−1.10–0.05)**	4,451	−0.10 (−0.56–0.37)
6. Hip fracture	4,497	0.65 (−0.02–1.33)	4,454	**0.74 (0.14–1.35)**
7. Emergency room visit	4,497	0.60 (−0.16–1.36)	4,454	0.65 (−0.03–1.33)
8. Other fracture	4,497	0.06 (−0.84–0.95)	4,454	0.04 (−0.75–0.84)
**b. SRM**				
	**Unadjusted**		**Adjusted^a^**	
**Single stressor**	***n***	**Regression coefficient (95% CI)**	***n***	**Regression coefficient (95% CI)**
1. Conflict	3,704	**1.83 (1.56–2.10)**	3,681	**1.09 (0.86–1.32)**
2. Falls	3,705	**0.95 (0.67–1.22)**	3,681	**0.61 (0.37–0.85)**
3. Major life stressor	3,704	**1.15 (0.87–1.43)**	3,681	**0.97 (0.73–1.22)**
4. Hospital stay	3,705	0.02 (−0.38–0.43)	3,681	**0.45 (0.10–0.80)**
5. Inpatient acute care	3,702	0.08 (−0.39–0.55)	3,678	**0.47 (0.06–0.87)**
6. Hip fracture	3,705	**0.67 (0.06–1.28)**	3,681	0.50 (−0.03–1.03)
7. Emergency room visit	3,705	0.40 (−0.31–1.12)	3,681	0.35 (−0.27–0.98)
8. Other fracture	3,705	0.73 (−0.02–1.49)	3,681	0.59 (−0.06–1.24)

*Statistically significant regression coefficients are bolded*.

*DRS, Depressive Rating Scale; SRM, Self-reported Mood scale*.

*In all models, facility was included as a second level*.

a *Adjusted for age, gender, length of stay, number of somatic diagnoses, the presence of a psychiatric diagnosis, cognitive functioning, ADL functioning, and social involvement*.

#### Adjusted Model With Outcome DRS

Conflict clearly had the strongest association with the observer-reported mood symptoms, on average, those with conflict had a DRS score that was 3 points higher than those without (regression coefficient = 3.07). The second largest adjusted regression coefficient was 1.46 for major life stressor. The association with conflict was greater in females than in males and greater in those with no to mild cognitive impairment than those with at least moderate impairment. Falls and hip fracture were also significantly associated with observer-reported mood symptoms. Upon stratification, the association with hip fracture remained significant in women, but not in men. Hospital stay and inpatient acute care had a negative association with DRS only in the unadjusted models.

#### Adjusted Model With Outcome SRM

Conflict and major life stressor also had the strongest association with self-reported mood symptoms. The adjusted regression coefficients were similar, 1.09 and 0.98, respectively. Again, the association between conflict and mood symptoms was greater in females and those with no to mild cognitive impairment. Falls, hospital stay, and inpatient acute care were also positively associated with self-reported mood.

The findings of the sensitivity analyses employing binary logistic regression to explore the association with the dichotomized outcomes were similar (Supplementary Table E-2).

### Combination of Stressors

Zero, one, two, and three or more stressors were reported for 45%, 33%, 15%, and 7% of the residents, respectively. Any number of stressors was associated with more mood symptoms than no stressors. Multiple stressors had a stronger association with mood on both outcomes than one stressor (Table 4). Supplementary Table E-3 provides the similar results of the binary logistic regressions examining the relationship between the number of stressors and the dichotomized outcomes. Supplementary Tables E-4, E-5 present the prevalence of the most common combinations of two stressors and their association with the mood outcomes.

**Table 4:** Overview of the association between the presence of one or more stressors and: a. observer-reported mood (DRS); b. self-reported mood (SRM) in the subpopulation with SRM complete.

**a. DRS**		
**Number of stressors (reference = 0)**	**Unadjusted regression coefficient (95% CI)**	**Adjusted^a^ coefficient (95% CI)**
	***n* = 4,493**	***n* = 4,451**
1	**1.96 (1.69–2.24)**	**1.66 (1.42–1.91)**
2	**3.00 (2.64–3.35)**	**2.59 (2.28–2.90)**
3 or more	**2.76 (2.29–3.22)**	**2.59 (2.17–3.00)**
**b. SRM**		
**Number of stressors** **(reference** **=** **0)**	**Unadjusted regression** **coefficient** **(95% CI)**	**Adjusted^a^ regression** **coefficient** **(95% CI)**
	***n*** **=** **3,701**	***n*** **=** **3,678**
1	**1.22 (0.95–1.48)**	**0.82 (0.59–1.05)**
2	**1.81 (1.48–2.15)**	**1.32 (1.01–1.59)**
3 or more	**1.98 (1.55–2.41)**	**1.63 (1.25–2.00)**

*Statistically significant regression coefficients are bolded*.

*DRS, Depressive Rating Scale; SRM, Self-reported Mood scale*.

*In all models, facility was included as a second level*.

a *Adjusted for age, gender, length of stay, number of somatic diagnoses, the presence of a psychiatric diagnosis, cognitive functioning, ADL functioning, and social involvement*.

## Discussion

### Single Stressors

Major life stressor and conflict with staff and/or other care recipients were the most commonly occurring stressors and were also most strongly associated with both mood outcomes. These stressors are therefore particularly suited for use in resilience research within the psychological domain in older residents of LTCFs. Falls are also unambiguously and significantly associated with both mood outcomes and may therefore be considered a stressor within the psychological domain. Events related to acute health issues or health care, such as inpatient/outpatient acute hospital care and fractures, were less strongly associated or not associated with mood outcomes in this population.

As mentioned, Garms-Homolová et al. recently reported a significant association of major life stressor in the last 90 days with DRS score in home care patients (13). More generally, the association between stressful life events and mood symptoms in older adults has been described repeatedly (29, 30).

The importance of conflict with care staff and/or other care recipients as a stressor in LTCF residents is a more novel finding. Not only did conflict have a strong association with mood symptoms, it is also the most prevalent (24%) stressor in this study. Although conflict has been acknowledged as an important point of attention within LTCF policy (31, 32), little empirical research is available on the topic (14). O'Rourke et al. described a positive association between conflict with staff and residents and sadness in LTCF residents in Canada with moderate (and severe) dementia (14). Interestingly, the prevalence of conflict was much lower in the Canadian population. Conflict with staff was described in 6% and with another care recipient in 7% of the residents, compared to 23% and 27%, respectively, of the residents with moderate to severe dementia in the current study. Characteristics such as age, gender, and length of stay were similar in the two (sub-)populations. The discrepancy may be a result of interpretation of conflict, cultural differences, or differences in characteristics of the LTCFs (for example, staffing levels and group activities within the LTCF).

The results of this study suggest that conflict is strongly associated with mood symptoms for older LTCF residents irrespective of their cognitive status. In an attempt to specify the stressor conflict further, we explored the association of “conflict with or repeated criticism of staff” (prevalence 16%) and “conflict with or repeated criticism of care recipient” (prevalence 18%) separately in Supplementary Material. The regression coefficients did not significantly differ from each other or from the coefficient for the combined stressor conflict with staff and/or care recipient (results not shown).

Future research should explore the impact of conflict on mood and quality of life of residents, both quantitatively and qualitatively. Subsequently, if relevant, research can focus on possibilities to prevent and resolve conflict with other residents as with staff at both the individual and LTCF policy level (14). Different nursing home conflict prevention strategies have been proposed previously, such as training staff to handle provocations and recognize inter-resident conflict, rotating staff responsibility for “difficult” residents, and facilitating open communication between staff and management (32). For now, this studies' results may motivate LTCF care providers and staff to be extra alert to resident conflict (both with other residents as with staff) and its consequences.

The association between conflict and observer-reported mood was particularly high. Possibly, the strong association is a result of the fact that items within the DRS may also be indicative of conflict, e.g., “persistent anger with self or others” and “made negative statements”. The SRM, on the other hand, only includes self-reported feelings of sadness, loss of interest, and anxiousness. To explore this possibility, sensitivity analyses were performed in which the association between conflict and an adapted DRS score in which the items that were theoretically also strongly indicative of conflict were removed. The two items “persistent anger with self or others” and “made negative statements” were removed, and an adapted score was calculated from a total of five items, leading to a maximum total score of 10. Removing these items only had a slight impact on the strength of the association between conflict and DRS (results not shown). Therefore, a theoretical overlap does not appear to explain the strong association between conflict and DRS.

Because, on average, major life stressor and conflict were negatively associated with mood, they are suited for resilience research (4, 6). In a next step, a longitudinal operationalization of resilience may involve having relatively little/no extra mood symptoms despite having undergone these stressors. Subsequently, individual and social factors that are associated with this resilience in the face of these stressors can be identified.

### Combination of Stressors

Multiple stressors were more strongly associated with both observer-reported and self-reported mood than one stressor. However, unlike in the study by Hildon et al., there does not seem to be an additive effect of more stressors, as three or more stressors were not significantly more associated with the mood outcomes than two stressors (6). The combination of major life stressor and conflict had the strongest association with both mood outcomes. Overall, the prevalence of the combination of stressors and the association between the most common combinations and the mood outcomes were in line with the findings on single stressors.

### Objective vs. Subjective Outcome

The associations between stressor and mood outcomes based on observer-report (objective) and self-report (subjective) in older LTCF residents were similar. Exceptions are the strength of the association with conflict as described above and the associations with hospital stay/inpatient acute care. People who were hospitalized had more self-reported depressive symptoms, although they tended to have less observer reported symptoms (DRS). A possible explanation for this contradiction may be that, in The Netherlands, the most (cognitively) frail residents are not always referred to hospital. Residents with cognitive impairment have higher scores on the DRS than the cognitively intact residents. The fact that the stressor occurs more often in the cognitively intact may have led to a lower DRS score in those who experienced hospitalization. As the residents who completed the SRM were more cognitively intact than the people without SRM scores, this effect may be less evident in the models with SRM.

### Strengths and Limitations

This study gives a first insight into stressors for LTCF residents, using both objective and subjective outcome measures in a large representative cohort of LTCF residents in The Netherlands. The use of, nearly complete, routine care data minimizes selection bias.

There are also limitations to consider. As this is a cross-sectional study, we cannot make inferences on the directionality/causality of the associations between the stressors and mood symptoms. For example, in the case of conflict, it is conceivable that the relationship is bidirectional.

This information on the possible stressors is limited by the information available within the interRAI assessment. For example, the descriptions of major life stressor are quite broad, resulting in limited knowledge on the exact nature of the stressor experienced. Other studies have described similarly broad stressors (6). Research utilizes more detailed questionnaires of major life type stressors, such as the List of Threatening Experiences Questionnaire (LTE-Q), and qualitative methods are of added value when studying experienced life stressors as they allow for a more detailed exploration of number, nature, and complexity of the stressors (30).

The interRAI dataset uniquely allows for comparison of the association with both observer-reported and self-reported mood outcomes. The models with the outcome SRM only apply for those capable/willing to answer the self-report questions (missing data not at random). On average, this population was slightly older, less cognitively, and functionally impaired and had less diagnoses and a considerably lower score on the DRS. Although inherent to this outcome type, this should be considered when interpreting the results. An example is discussed in Section Objective vs. Subjective Outcome.

## Conclusions

Major life stressor and conflict had the strongest association with both mood outcomes and are, therefore, particularly suited as stressors within psychological resilience research in older LTCF residents.

The association between conflict with other residents/care staff and mood symptoms was remarkably strong. Further (longitudinal) research is necessary to determine the directionality and relevance of this association for LTCF practice.

## Data Availability Statement

The data analyzed in this study is subject to privacy restrictions. Requests to access these datasets should be directed to HH (hpj.vanhout@amsterdamumc.nl).

## Ethics Statement

Ethical review and approval was not required for the study on human participants in accordance with the local legislation and institutional requirements. Written informed consent for participation was not required for this study in accordance with the national legislation and the institutional requirements.

## Author Contributions

MA, HH, MS, AB, CH, and KJ participated in the initial selection of stressors. MA performed the necessary data management, cleaning, and drafted the manuscript. MA and JT performed data analysis. All authors contributed to the conception and design of the article and finalization of the manuscript.

## Funding

This work was supported by Amsterdam UMC, Amsterdam, The Netherlands.

## Conflict of Interest

The authors declare that the research was conducted in the absence of any commercial or financial relationships that could be construed as a potential conflict of interest.

## Publisher's Note

All claims expressed in this article are solely those of the authors and do not necessarily represent those of their affiliated organizations, or those of the publisher, the editors and the reviewers. Any product that may be evaluated in this article, or claim that may be made by its manufacturer, is not guaranteed or endorsed by the publisher.
