# Supplementary material for: The Association Between Possible Stressors and Mood Outcomes in Older Residents of Long-Term Care Facilities
Source: Front Psychiatry. 2022 Apr 4;13:811252. doi: 10.3389/fpsyt.2022.811252 (PMC9015094; doi:10.3389/fpsyt.2022.811252)
Supplement: Supplementary file 1 [file Data_Sheet_1.PDF]

Supplementary Material

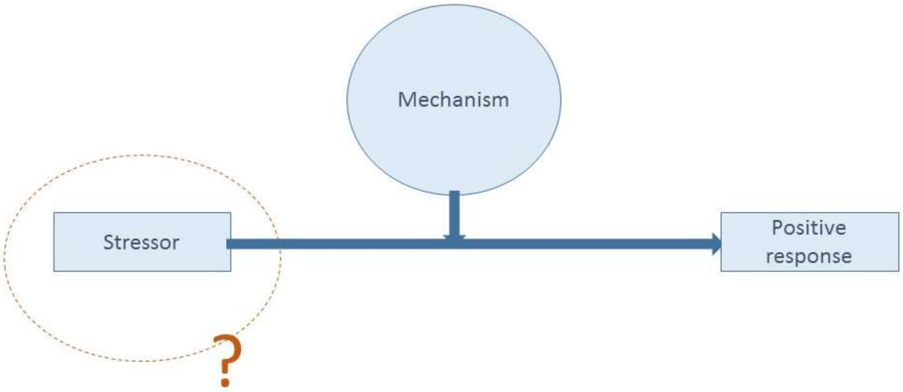

Figure E-1: Resilience. Resilience encompasses the presence of a positive response to some type of stressor (adversity) and the mechanism by which that positive response is achieved. In the current study we aim to identify important stressors in long term care facilities by determining which possible stressors have the strongest negative relationship with mood outcomes in the complete population.

Table E-1: Statistically significant stratifications within the association of the single stressors with: a. observer-reported mood (DRS); and b. self-reported mood (SRM) in the subpopulation with SRM complete. Statistically significant regression coefficients are bolded.

**a. DRS**			
			**Adjusted[a]**
**Single stressor**	**stratification**	**n**	**Regression coefficient (95% CI)**
Conflict	C: intact	3067	**3.26 (2.96-3.55)**
	C: impaired	1387	**2.53 (2.14-2.92)**
	G: male	1304	**2.50 (2.08-2.93)**
	G: female	3150	**3.28 (3.00-3.57)**
Hip fracture	G: male	1304	-0.38 (-1.45-0.68)
	G: female	3150	**1.18 (0.45-1.91)**
Inpatient acute care	C: intact	3067	0.08 (-0.43-0.58)
	C: impaired	1384	-1.01 (-2.08-0.05)

**b. SRM**			
			**Adjusted[a]**
**Single stressor**	**stratification**	**n**	**Regression coefficient (95% CI)**
Conflict	C: intact	2857	**1.31 (1.01-1.61)**
	C: impaired	824	**0.75 (0.36-1.13)**
	G: male	1070	**0.63 (0.14-1.12)**
	G: female	2611	**1.26 (1.00-1.53)**

Note: DRS=Depressive Rating Scale, SRM=Self-reported Mood scale, C: intact= no to mild cognitive impairment, sCPS <3; C: impaired= moderate to severe cognitive impairment, sCPS ≥3; G= gender.
In all models facility was included as a second level.
[a] Adjusted for age, gender, length of stay, number of somatic diagnoses, the presence of a psychiatric diagnosis, cognitive functioning, ADL functioning, and social involvement.

Table E-2: Odds ratios (OR's) of falling above the cut off point for mood symptoms for those who experienced the single stressors as compared to those whom did not: a. observer-reported mood (DRS), b. self-reported mood (SRM) in the subpopulation with SRM complete. Statistically significant OR's are bolded.

**a. DRS**				
		**Unadjusted**		**Adjusted[a]**
**Single stressor**	**n**	**OR (95% CI)**	**n**	**OR (95% CI)**
Conflict	4496	**5.31 (4.56-6.19)**	4454	**4.93 (4.19-5.81)**
Falls	4497	**1.54 (1.32-1.79)**	4454	**1.42 (1.21-1.68)**
Major life stressor	4496	**1.98 (1.69-2.32)**	4454	**2.09 (1.76-2.49)**
Hospital stay	4497	0.82 (0.65-1.03)	4454	1.03 (0.80-1.32)
Inpatient acute care	4494	0.85 (0.65-1.11)	4451	1.03 (0.77-1.38)
Hip fracture	4497	1.35 (0.97-1.88)	4454	**1.51 (1.04-2.19)**
Emergency room visit	4497	1.33 (0.91-1.93)	4454	1.42 (0.94-2.14)
Other fracture	4497	1.10 (0.70-1.71)	4454	1.11 (0.68-1.81)
**b. SRM**				
		**Unadjusted**		**Adjusted[a]**
**Single stressor**	**n**	**OR (95% CI)**	**n**	**OR (95% CI)**
Conflict	3704	**2.76 (2.32-3.27)**	3681	**2.04 (1.69-2.47)**
Falls	3705	**1.66 (1.39-1.97)**	3681	**1.50 (1.24-1.82)**
Major life stressor	3704	**1.86 (1.56-2.22)**	3681	**1.94 (1.58-2.37)**
Hospital stay	3705	1.03 (0.80-1.33)	3681	**1.41 (1.06-1.88)**
Inpatient acute care	3702	0.90 (0.67-1.23)	3678	1.17 (0.83-1.64)
Hip fracture	3705	**1.50 (1.04-2.17)**	3681	**1.61 (1.04-2.50)**
Emergency room visit	3705	1.29 (0.83-2.00)	3681	1.33 (0.80-2.21)
Other fracture	3705	**1.64 (1.03-2.61)**	3681	**1.75 (1.03-3.00)**

Note: DRS=Depressive Rating Scale, SRM=Self-reported Mood scale.
In all models facility was included as a second level.
[a] Adjusted for age, gender, length of stay, number of somatic diagnoses, the presence of a psychiatric diagnosis, cognitive functioning, ADL functioning, and social involvement.

Table E-3: Odds ratios (OR's) of falling above the cut off point for mood symptoms for those who experienced 1 or more stressors as compared to those whom did not: a. observer-reported mood (DRS) b. self-reported mood (SRM) in the subpopulation with SRM complete. Statistically significant OR's are bolded.

**a. DRS**		
**Number of stressors (reference=0)**	**Unadjusted OR (95% CI)**	**Adjusted[a] OR (95% CI)**
	**n=4493**	**n=4451**
1	**2.37 (2.04-2.75)**	**2.34 (1.99-2.76)**
2	**3.82 (3.15-4.62)**	**3.92 (3.18-4.83)**
3 or more	**3.37 (2.63-4.32)**	**3.82 (2.91-5.01)**
**b. SRM**		
**Number of stressors (reference=0)**	**Unadjusted OR (95% CI)**	**Adjusted[a] OR (95% CI)**
	**n=3701**	**n=3678**
1	**2.02 (1.70-2.42)**	**1.76 (1.45-2.14)**
2	**2.64 (2.12-3.29)**	**2.32 (1.81-2.97)**
3 or more	**3.06 (2.31-4.04)**	**3.11 (2.27-4.26)**

Note: DRS=Depressive Rating Scale, SRM=Self-reported Mood scale.
In all models facility was included as a second level.
[a] Adjusted for age, gender, cognition, ADL functioning, social involvement, number of somatic diagnoses, presence of psychiatric diagnoses, length of stay.

Table E-4: Overview of the prevalence of the most common combination of stressors and their association with: a. observer-reported mood (DRS); b. self-reported mood (SRM) in the subpopulation with SRM complete. Statistically significant regression coefficients are bolded.

a. DRS

				Unadjusted		Adjusted[a]
**Combination of stressors**	**Freq.**	**Perc. (%)**	**n**	**Regression coefficient (95% CI)**	**n**	**Regression coefficient (95% CI)**
1.Conflict & falls	274	6.1	4496	**3.61 (3.13-4.09)**	4454	**2.90 (2.47-3.33)**
2. Hospital stay & inpatient acute care	241	5.4	4494	**-0.57 (-1.14— 0.01)**	4451	-0.10(-0.61-0.40)
3. Major life stressor & conflict	224	5.0	4496	**4.13 (3.61-4.65)**	4454	**3.67 (3.21-4.13)**
4. Major life stressor & falls	222	4.9	4496	**2.48 (1.94-3.02)**	4454	**2.09 (1.61-2.57)**
5. Hospital stay & major life stressor	153	3.4	4496	**0.87 (0.19-1.55)**	4454	**1.15 (0.55-1.76)**
6. Major life stressor & inpatient acute care	119	2.6	4493	**0.81 (0.05-1.57)**	4451	**1.14 (0.46-1.81)**
7. Hospital stay & falls	118	2.6	4497	0.22 (-0.56-1.01)	4454	0.59 (-0.11-1.29)

b. SRM

		Unadjusted		Adjusted[a]
**Combination of stressors**	**n**	**Regression coefficient (95% CI)**	**n**	**Regression coefficient (95% CI)**
1. Conflict & falls	3704	**2.15 (1.68-2.62)**	3681	**1.46 (1.06-1.86)**
2. Hospital stay & inpatient acute care	3702	0.04 (-0.47-0.55)	3678	0.43 (-0.01-0.88)
3. Major life stressor & conflict	3704	**2.23 (1.75-2.72)**	3681	**1.65 (1.24-2.07)**
4. Major life stressor & falls	3704	**1.52 (1.03-2.01)**	3681	**1.12 (0.70-1.54)**
5. Hospital stay & major life stressor	3704	**0.83 (0.22-1.45)**	3681	**0.93 (0.41-1.46)**
6. Major life stressor & inpatient acute care	3701	**0.88 (0.2 0-1.56)**	3678	**1.00 (0.42-1.58)**
7. Hospital stay & falls	3705	**0.76 (0.05-1.46)**	3681	**0.89 (0.28-1.49)**

Note: DRS=Depressive Rating Scale, SRM=Self-reported Mood scale.
In all models facility was included as a second level.
[a] Adjusted for age, gender, length of stay, number of somatic diagnoses, the presence of a psychiatric diagnosis, cognitive functioning, ADL functioning, and social involvement.

Table E-5: Odds ratios (OR's) of falling above the cut off point for mood symptoms for those who experienced combination of stressors as compared to those whom did not: a. observer-reported mood (DRS) b. self-reported mood (SRM) in the subpopulation with SRM complete. Statistically significant OR's are bolded.

a. DRS

	Unadjusted		Adjusted[a]	
**Single stressor**	**n**	**OR (95% CI)**	**n**	**OR (95% CI)**
1. Conflict & falls	4496	**5.57 (4.18-7.42)**	4454	**5.02 (3.72-6.80)**
2. Hospital stay & inpatient acute care	4494	0.88 (0.66-1.17)	4451	1.07 (0.78-1.47)
3. Major life stressor & conflict	4496	**7.28 (5.23-10.14)**	4454	**7.78 (5.49-11.02)**
4. Major life stressor & falls	4496	**2.61 (1.97-3.46)**	4454	**2.54 (1.87-3.44)**
5. Hospital stay & major life stressor	4496	1.29 (0.92-1.82)	4454	**1.54 (1.07-2.24)**
6. Major life stressor & inpatient acute care	4493	1.35 (0.92-1.98)	4451	**1.66 (1.10-2.50)**
7. Hospital stay & falls	4497	1.11 (0.75-1.64)	4454	1.36 (0.89-2.07)

b. SRM

	Unadjusted		Adjusted[a]	
**Single stressor**	**n**	**OR (95% CI)**	**n**	**OR (95% CI)**
1. Conflict & falls	3704	**2.86 (2.12-3.87)**	3681	**2.27 (1.63-3.16)**
2. Hospital stay & inpatient acute care	3702	0.98 (0.70-1.36)	3678	1.28 (0.89-1.85)
3. Major life stressor & conflict	3704	**3.57 (2.61-4.88)**	3681	**3.25 (2.30-4.60)**
4. Major life stressor & falls	3704	**2.19 (1.61-2.98)**	3681	**2.08 (1.48-2.92)**
5. Hospital stay & major life stressor	3704	**1.75 (1.20-2.55)**	3681	**2.18 (1.43-3.32)**
6. Major life stressor & inpatient acute care	3701	1.45 (0.95-2.22)	3678	**1.75 (1.10-2.80)**
7. Hospital stay & falls	3705	**1.66 (1.08-2.54)**	3681	**2.05 (1.26-3.32)**

Note: DRS=Depressive Rating Scale, SRM=Self-reported Mood scale.
In all models facility was included as a second level.
[a]Adjusted for age, gender, length of stay, number of somatic diagnoses, the presence of a psychiatric diagnosis, cognitive functioning, ADL functioning, and social involvement.
